# The Role of Primate Prefrontal Cortex in Bias and Shift Between Visual Dimensions

**DOI:** 10.1093/cercor/bhz072

**Published:** 2019-04-10

**Authors:** Farshad A Mansouri, Mark J Buckley, Daniel J Fehring, Keiji Tanaka

**Affiliations:** 1 Cognitive Neuroscience Laboratory, Department of Physiology, Monash Biomedicine Discovery Institute, Monash University, Victoria, Australia; 2 ARC Centre of Excellence for Integrative Brain Function, Monash University, Victoria, Australia; 3 Department of Experimental Psychology, Oxford University, Oxford, UK; 4 Cognitive Brain Mapping Laboratory, RIKEN Center for Brain Science, Wako, Saitama, Japan

**Keywords:** attentional set shifting, lesion-behavioral study, prefrontal cortex, visual dimensions

## Abstract

Imaging and neural activity recording studies have shown activation in the primate prefrontal cortex when shifting attention between visual dimensions is necessary to achieve goals. A fundamental unanswered question is whether representations of these dimensions emerge from top-down attentional processes mediated by prefrontal regions or from bottom-up processes within visual cortical regions. We hypothesized a causative link between prefrontal cortical regions and dimension-based behavior. In large cohorts of humans and macaque monkeys, performing the same attention shifting task, we found that both species successfully shifted between visual dimensions, but both species also showed a significant behavioral advantage/bias to a particular dimension; however, these biases were in opposite directions in humans (bias to color) versus monkeys (bias to shape). Monkeys’ bias remained after selective bilateral lesions within the anterior cingulate cortex (ACC), frontopolar cortex, dorsolateral prefrontal cortex (DLPFC), orbitofrontal cortex (OFC), or superior, lateral prefrontal cortex. However, lesions within certain regions (ACC, DLPFC, or OFC) impaired monkeys’ ability to shift between these dimensions. We conclude that goal-directed processing of a particular dimension for the executive control of behavior depends on the integrity of prefrontal cortex; however, representation of competing dimensions and bias toward them does not depend on top-down prefrontal-mediated processes.

## Introduction

In attentional set-shifting tasks such as the Stroop test and the Wisconsin Card Sorting Test (WCST), allocation and shift of attention between different stimulus dimensions are required (Mansouri and Tanaka 2002; Maunsell and Treue 2006; Mansouri et al. 2007; Buckley et al. 2009; Mansouri et al. 2009). In these tasks, attention is oriented, presumably by top-down control processes, to an abstract conceptual entity that is the sensory dimension of visual stimuli (e.g., color or shape dimensions). Previous imaging studies in humans and monkeys suggest that a distributed network of frontoparietal regions including dorsal and lateral parts of the prefrontal cortex supports the allocation and shift of selective attention to and between sensory dimensions (Konishi et al. 1998; Monchi et al. 2001; Nakahara 2002; Paneri and Gregoriou 2017). Activation in anterior cingulate cortex (ACC) has also been observed in various tasks that demand top-down executive control of goal-directed behavior, suggesting that ACC also contributes to allocation and reorientation of attentional resources (Mansouri et al. 2017). Neuropsychological examination of patients (Stuss et al. 2000; Boschin et al. 2017) and lesion-behavioral studies in monkeys (Dias et al. 1996; Buckley et al. 2009; Mansouri et al. 2014) have indicated the crucial involvement of dorsolateral prefrontal cortex (DLPFC), ACC, and orbitofrontal cortex (OFC) in tasks requiring allocation to, and shifting attention from, sensory dimensions. Recording neuronal activity in the DLPFC, ACC, and OFC in the context of attentional set-shifting tasks has shown that neuronal activity in these regions encode several distinct aspects of the task including the relevant sensory dimension (Mansouri et al. 2007; Kuwabara et al. 2014; Mansouri et al. 2014). Together, these studies suggest an important role for medial frontal and prefrontal cortical regions in attention to dimensions and associated rule-guided behavior therein.

In the context of tasks that require dimension-based matching, for example, matching-by-color or matching-by-shape, toward visual stimuli, humans have repeatedly been shown to have a behavioral advantage/bias in allocating attention to, and redirecting attention away from, particular sensory dimensions. The direction of some observed biases are age dependent, suggesting an association with developmental changes of underlying brain networks (Grant et al. 1949; Grant and Curran 1952; Otto and Askov 1968; Brown and Campione 1971; Prevor and Diamond 2005). Such dimension-based biases may appear in children and may influence their performance (Ellefson et al. 2006), and saccadic eye movements (Yi et al. 2012), in dimensional set-shifting tasks. Intriguingly, children with autism spectrum disorders underperform compared with controls; moreover, they do not appear to show any dimension-based bias (Yi et al*.* 2012). Other studies also suggest age dependency of these kinds of bias and altered patterns of such biases in some neurological disorders (Otto and Askov 1968; Ellefson et al*.* 2006). The links between these kinds of attentional biases and cognitive abilities supporting allocation and shift of attention and their relations to neurodevelopmental disorders are scientific unknowns that warrant further investigation. Importantly, it remains unclear whether such dimension-based biases result from top-down attentional modulation, mediated through prefrontal cortical regions, or from processing advantages in early stages of visual information processing.

Macaque monkeys are frequently used as animal models for determination of the neural substrates and mechanisms underlying cognitive control and selective attention as there are broad similarities in the structure and organization of the visual system between humans and macaques (Tanaka 1997; Van Essen et al. 2001; Orban et al. 2004). There are also significant similarities in the cytoarchitectonic organization of human and macaque prefrontal cortex (Petrides and Pandya, 1999; Petrides et al. 2005, 2012; Mackey and Petrides, 2010). However, it is still unclear whether monkeys manifest a bias toward particular sensory dimensions in attentional set-shifting tasks in a similar way to humans. To examine the neural bases of bias and attentional shifts between sensory dimensions, we trained a large cohort of macaque monkeys and humans to perform a matching task that required selective attention to the color or shape dimension of visual stimuli with the relevant dimension periodically changing without notice. Therefore, the task required cognitive flexibility to detect changes in environmental/contextual demands (signaled only by the presence or absence of visual feedback and reward delivery for correct matching choices) and the ability to efficiently shift attention between these 2 sensory dimensions. Importantly, testing humans and macaques in the context of the same task allowed us to examine to what extent any of their evident biases to a particular sensory dimension were similar in nature or degree. We hypothesized that if the emergence of visual dimensions and the bias toward them depend on top-down control interventions arising from higher stages of processing in the prefrontal cortical regions, then selective lesions within these areas would eliminate such dimension-based biases. However, we reasoned that if dimension-specific biases emerge in the early stages of visual processing, then these biases would remain intact after damage to prefrontal or medial frontal cortical regions. We found that although lesions in certain prefrontal or medial frontal cortical regions (DLPFC, OFC, and ACC) impaired shifting between dimensions, the dimension-based bias remained after lesions in many different prefrontal and medial frontal cortical regions. Together, this indicates that while parts of prefrontal and medial frontal cortex are essential for the cognitive flexibility in shifting between dimensions, these prefrontal regions are not necessary for the emergence of dimension-based bias.

## Materials and Methods

### Participants

Human participants: 85 participants (Monash University students) within the age range of 18–27 (22.12 ± 0.24; Mean ± standard error) joined the study as volunteers. Fifty-five (35 female, 20 male) and 30 participants (19 female, 11 male) performed the 2-rule and the 3-rule versions of the WCST analog, respectively. All procedures followed the guidelines stipulated by Monash University Human Research Ethics Committee.

Monkeys: 21 macaque monkeys [1 female (*Macaca fuscata)* and 20 males (14 *M. mulatta* and six *M. fuscata*)] were included in the study. Their weight at the time of surgery was between 6 to 9 Kg. Monkeys had no previous experience with any other task before learning the WCST analog. All experimental procedures followed the ethics guidelines stipulated by RIKEN Brain Science Institute or the Oxford University Animal Ethics Committee and UK Animals (Scientific Procedures) Act 1986.

### Behavioral Tasks

**Figure 1:**
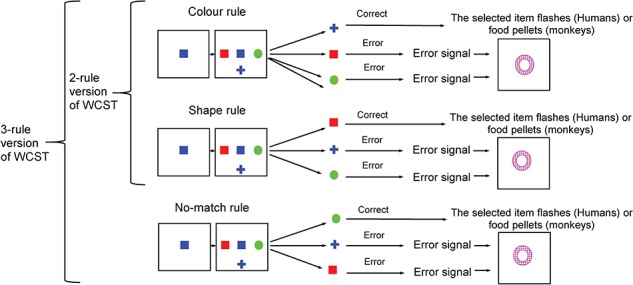
The Wisconsin Card Sorting analog. In the WCST used in monkeys, trials started by sample onset (the sample was selected randomly, without replacement, until the entire set of 36 different samples, made of 6 colors and 6 shapes, was used) and after the monkey touched the sample and released its hand; 3 test items appeared surrounding the sample. The monkeys had to match the sample to 1 of 3 test items either by color or by shape and touch the correct item within a response window to receive a reward. In the WCST for humans, each trial started by presentation of a Start cue (a large gray color circle) at the center of the touchscreen. Pressing a switch turned off the Start cue and led to the sample onset. The rest of the trial events was similar for monkeys and humans. A reward or a distinct visual error signal (white annulus) was given as feedback after correct or erroneous matching, respectively. The relevant rule for matching (matching by shape or matching by color) was consistent within a block of trials, and it changed without any notice when monkeys or humans achieved the shift criterion.

Humans and monkeys performed computerized versions of the WCST analog. The main task shown in Figure 1 has been validated in previous studies (Mansouri and Tanaka, 2002; Buckley et al. 2009; Mansouri et al*.* 2007, 2014; Kuwabara et al. 2014; Mansouri et al. 2015, Mansouri et al. 2016; Boschin et al. 2017; Mansouri et al. 2017). In the WCST analog used for monkeys, in each trial, first the sample was shown at the center of the touchscreen, and after the monkey touched the sample, 3 test items appeared surrounding the sample. In the WCST used for humans, in each trial, first a start cue appeared and instructed the subjects to initiate the trial by pressing a switch. Pressing the switch led to the onset of a sample, and if the subjects kept pressing the switch down, 3 test items appeared surrounding the sample (at the left, right, and bottom). The events after the sample onset were the same in the WCST analogs used for monkeys and humans. In each trial, subjects had to select and touch 1 of the 3 test items that matched the sample based on the currently relevant dimension for matching (color or shape). The subjects had to find the relevant dimension by trial and error and match other visual stimuli based on that dimension in the following trials. The relevant dimension was not cued and changed, unannounced, when the subjects achieved a shift criterion. The relevant sensory dimension in the first block of each daily session alternated between color and shape across the daily sessions. The performance criterion that when attained shifted the dimension was 17 correct choices in 20 consecutive trials (i.e., 85% correct across 20 trials) for monkeys and 9 correct choices out of 10 consecutive trials for humans (i.e., 90% correct across 10 trials). The versions of the WCST analog used in this study utilized 36 different stimuli, which were composed of 6 colors (red, yellow, green, cyan, blue, and magenta) and 6 shapes (square, triangle, circle, hexagon, cross, and ellipse). The sample in each trial was selected at random (without replacement until the entire set had been used) from the 36 stimuli. In each trial, the test items were also selected from the same set of 36 stimuli and at random (with the restrictions imposed by the necessity to generate either a congruent or incongruent trial). The locations of the 3 test items (i.e., to the left/right/bottom of the sample) were also chosen at random. The sizes of the stimuli were 5–6 cm on the screen. The center-to-center distance between the test items and sample was 15 cm on the screen.

In the 2-rule version of the WCST, only 2 potential dimensions for matching were included (color or shape dimensions) (Fig. 1). The 3-rule version was similar with the 2-rule version of the WCST; however, an additional no-match rule (selecting the target that did not match by either color or shape dimension) was also included. Addition of no-match rule allowed examining shifting between 3 separate rules using the same visual stimuli. Participants performed 9 blocks in the 3-rule version (3 color, 3 shape, and 3 no-match blocks), and these blocks were pseudorandomly ordered so that a rule was not repeated in consecutive blocks and became relevant only after the participants achieved criterion with the other 2 alternative rules. The same set of stimuli (36 items made of 6 colors and 6 shapes) was used in the 2-rule and 3-rule versions of the computerized WCST.

Humans and monkeys also performed another version of the 2-rule WCST analog (WCST conflict) in which the level of conflict between dimensions varied trial by trial by inclusion of congruent and incongruent trials. This specific version of the task has also been validated previously (Mansouri et al. 2002, 2007). In incongruent trials, one of the test items matched the central sample in shape, another test item matched the central sample in color, and the third test item did not match the sample in either shape or color. In congruent trials, 1 of the test items matched the sample in both shape and color, and the other 2 test item did not match the sample in either shape or color, and therefore, there was no conflict between the matching rules. Incongruent and congruent trials were intermingled randomly within a block. The standard version of the WCST (only incongruent trials) and the WCST-conflict (congruent and incongruent trials randomly intermingled) were both run in the same daily session for humans, but these 2 task variants were run on separate series of days for monkeys. A food pellet (190 mg; BioServe) was given to monkeys in reward for each correct trial, and they got access to an automated lunch box (filled with monkeys’ daily food/fruits and positioned in front of the monitor) that opened immediately at the end of each testing session. To encourage a speeded response, a response time window was considered for humans (900 ms) and for monkeys (3000 ms). The combined motivational factors of the trial-by-trial expected food pellets and the end-of-session lunch encouraged the monkeys to respond with good accuracy and also rapidly; the longer response window was implemented for monkeys considering their distance from the screen and to avoid consecutive timeout trials (no reward trials) that occur more frequently with short response windows, which could be discouraging for monkeys. In humans, data from 2 blocks of testings (with a 10-min rest period in between) within a daily session were included in the data analyses. Monkeys learned to perform the 2-rule version of the WCST in about 10–12 months. After monkeys’ performance in the WCST reached a plateau, 15 sessions of the WCST were collected in which the animals completed 300 trials per session. All 21 monkeys were tested with the standard WCST; the WCST-conflict was tested in 13 monkeys (10 *M. mulatta* and 3 *M. fuscata*).

Whereas humans received a structured explanation about the task, rules, and procedures and practiced the task before initiation of data collection, monkeys were first pre-trained in a delayed matching-to-sample task to match multi-color and multi-form visual items and after acquiring the general matching rule proceeded to then apply the general matching rule to simple visual stimuli as used in the WCST analog. Subsequently, they were trained for more specific dimension-based matching based on color or shape matching rules in separate daily sessions. Initially, no shift was required within these sessions, but as the animals progressed, they transferred to training in sessions where shifts between the 2 matching rules were introduced within a session. Monkeys were trained for many months achieving a high performance level with each dimension (85% correct). Accordingly, they had experience shifting hundreds of times between color and shape dimensions and therefore were highly trained in matching with and shifting between color and shape dimensions by the time the 15 sessions of data were acquired for analysis in this project. In these 15 sessions, the first relevant dimension in each daily testing session was altered day by day, and monkeys received the same type and amount of reward for performing the task, and therefore, there were no nonspecific factors available to bias the monkeys’ performance toward one of the dimensions.

### Electrodermal Activity

We measured electrodermal activity (EDA, responses in skin conductance) of humans while they performed the 3-rule version of the WCST. An electrodermal recording unit (ML116 GSR Amp—ADInstruments) with PowerLab (26 T—ADInstruments) and an amplifier were used for recording EDA and also for monitoring and storage of data. During task performance, the skin conductance was continuously recorded (sampling rate of 75 kHz) by 2 metal electrodes attached to the palmar surface of the index and ring fingers of participant’s nondominant hand. Participants were instructed to avoid moving their nondominant hand and keep it on a pad over a desk. The skin conductance, measured in Standard International conductance units (microsiemens), was registered with event codes given by the behavioral control software (CORTEX from National Institute of Mental Health) to assess the event-related phasic changes in the EDA signal. The amplitude of phasic activity was determined as the difference between the maximum and minimum value (Boucsein 2012) within a 3-s window following an event (feedback to participant’s decision). Due to factors such as motion artifact, cold hand, or very low levels of sweating in some participants, the EDA response could not be reliably recorded in some of the sessions, and therefore, their data were excluded from the analyses. The data from 25 participants (14 females, 11 males) were used for the related analyses. Two observers blind to the results assessed and decided about inclusion or exclusion of the EDA records in each participant. In each participant, EDA values were normalized by dividing them by the mean of EDA in all conditions.

### Data Analyses and Statistical Approaches

In all the analysis of variance tests (ANOVA), we used raw data (percentage of correct responses, response time, or EDA) without any transformation or removing outliers. We did not remove any data points as outliers to avoid any arbitrary procedures being applied to exclude data. However, to facilitate comparison of data from different sessions and groups in our figures, response time values were normalized in each condition by dividing it by the mean response time in all the conditions in that session. Considering the differences in the level of sweating between participants, EDA data were also normalized following the same procedure. The effects of dimension and conflict on various behavioral measures were assessed by repeated-measure ANOVA. Mauchly’s Test was used to examine sphericity, and Greenhouse–Geisser correction was applied when necessary. Two-tailed *t-*test with Bonferroni adjustment for multiple comparisons was used for all pairwise comparisons. Data for each human participant (on WCST analog and WCST-conflict version) were collected in one daily session, and therefore, a single mean was used from each human participant for each relevant data analysis. However, because data for monkeys were collected in multiple sessions (i.e., WCST analog and WCST-conflict were performed in separate sessions), a “Monkey” factor was included in the ANOVA, and session means from each monkey were calculated for each relevant data analysis.

### Testing Setup

Humans: 3 participants were tested simultaneously while located in separate testing rooms. Recording and monitoring setups were in a separate “control room''. Participants’ behavior and hand movement were monitored through a video camera located in each testing room. CORTEX program was used to run the behavioral tests and record behavioral measures (at millisecond resolution). Participants read an explanatory statement about the task before coming to the test session and on the testing day received a structured briefing on the test procedure and requirement for matching based on the 3 rules (color, shape and no-match). The testing session started with 3 “practice blocks”, which made sure all participants understood the task requirement and procedure before data collection. The first practice block included only congruent trials, while the second and third practice blocks included only incongruent trials, requiring the application of the color or shape rule, respectively. Within these practice blocks, the shift criterion was set at 5 correct responses out of 5 consecutive trials. Following these practice blocks, participants performed 9 blocks of trials (3 colors, 3 shapes, and 3 no-match rules).

Monkeys: the tasks (Fig. 1) were provided in an automated test apparatus within a sound attenuated and well-ventilated cubicle. The monkey sat, with no restraint, in a wheeled test cage fixed in position in front of a touch-sensitive screen on which the stimuli were displayed. A computer, with a millisecond accuracy timer-card to record response times, controlled the experiment and conducted data acquisition. When the monkeys completed 300 trials, the testing session ended, and a lunch box was automatically opened and allowed the monkeys’ access to their daily lunch in the cognitive testing cubicle. Seven and 14 monkeys were trained and tested at Oxford University and RIKEN institute, respectively. The same software and criteria were used for training and testing the 21 monkeys.

### Lesion-behavioral Study

Surgery: the details of surgical approach and lesion extent have been reported in our previous studies (Mansouri et al*.* 2007; Buckley et al*.* 2009; Mansouri et al*.* 2014; Mansouri et al*.* 2015). Briefly, after completing prelesion data collection, monkeys had a rest for a week and then were operated under isoflurane anesthesia (1–2.75%, to effect, in 100% oxygen) while mechanically ventilated. After opening the skin and underlying tissues, the temporal muscles were retracted to expose the skull surface. A bone flap was removed, and the dura was cut and reflected to access the cortex. Using an operating microscope, a small-gauge metal aspirator attached to a controllable suction pump was used for making aspiration lesions. Aspiration was visually guided to complete the intended lesion extent. White matter under the cortex, which is easily distinguishable from gray matter, was spared as much as possible. Operated monkeys rested for approximately 14 days after surgery before starting the postlesion data collection. Unoperated control animals rested for the same period of time between preoperative and postoperative testing.

Intended lesion extent: all lesions were done bilaterally (Fig. 5). We used the cytoarchitectonic map of Petrides and Pandya (1999) for delineating the intended lesion extent in the prefrontal and medial frontal cortices. The intended lesion extent in DLPFC group included cortex in both banks and in the fundus of the Principal sulcus (Petrides and Pandya, 1999; Petrides et al. 2012). For the ACC lesion, a small-gauge metal aspirator was used to aspirate the cortex within the dorsal and ventral banks of the anterior cingulate sulcus (areas 24c, 24c′) (Vogt et al. 1987) in each hemisphere. The caudal limit of the lesion in the cingulate sulcus was at the level of the midpoint of the precentral dimple and the lesion extended rostrally for the full extent of the cingulate sulcus. The intended extent of the OFC lesion (Fig. 5) included the entire cortex between the medial and lateral orbital sulci. The lesion was extended to include the medial bank of the lateral orbital sulcus and medially until the lateral bank of the rostral sulcus. An imaginary line drawn between the anterior tips of the lateral and medial orbital sulci determined the anterior end of the lesion. The posterior extent was an imaginary line drawn just anterior to the posterior tips of these 2 sulci. Therefore, the intended lesion included cytoarchitectonic cortical areas 11, 13, and 14 on the orbital surface (Mackey and Petrides, 2010). The intended extent of the sdlPFC lesion included the cortex on the dorsolateral aspect of the prefrontal cortex starting 1 mm dorsal to the principal sulcus and extending dorsally up to the midline (i.e., lateral area 9 and the dorsal portions of areas 46 and 9/46) (Petrides and Pandya 1999) but excluding ventrally situated cortex that lay within the principal sulcus area; the lesion excluded posteriorly located areas 8A, 8 Bd, and 8Bv and did not extend anteriorly into area 10. For the frontal pole cortex (FPC) lesion, the caudal limits of the lesions were imaginary vertical lines drawn 2 mm posterior to the rostral end of the principal sulcus on the dorsal, medial, and orbital surfaces. All cortex on the most anterior part of the prefrontal cortex that were rostral to these limits on the dorsal, medial, and orbital surfaces was removed (Fig. 5).

Delineating lesion extent: structural magnetic resonance imaging (MRI) (4 Tesla) and histology were used to confirm the actual lesion extent (Mansouri et al*.* 2007; Buckley et al*.* 2009; Mansouri et al*.* 2015). For histological examination of the lesion extent, at the conclusion of the experiments, animals with lesions were deeply anesthetized and then perfused through the heart with saline followed by formol–saline solution. Their brains were removed from the skull and allowed to sink in sucrose–formalin solution. Then, 50 μm sections were cut using a frozen section method. Every 5th or 10th section was retained and stained with cresyl violet. For 2 DLPFC lesion animals, microscopic examination of the stained sections confirmed complete lesions of the entire anterior posterior extent of the cortex in both banks and the fundus of the principal sulcus with no damage outside of the intended region. For the 2 other monkeys with the DLPFC lesion (one female and one male), MRI confirmed the extent of the lesion in the intended area. The ACC lesions were also complete and within the intended boundaries, apart from in one macaque, ACC3, whose lesion was slightly larger than intended in one hemisphere, and in another macaque, ACC4, where the lesion did not extend quite as far posteriorly as in the other 3 animals in order to avoid damaging ascending branches of the anterior cerebral artery. The extent of lesions in OFC, sdlPFC, and the FPC was as intended.

## Results

### Performance of Monkeys and Humans in the 2-rule WCST (Only Incongruent Conditions)

**Figure 2:**
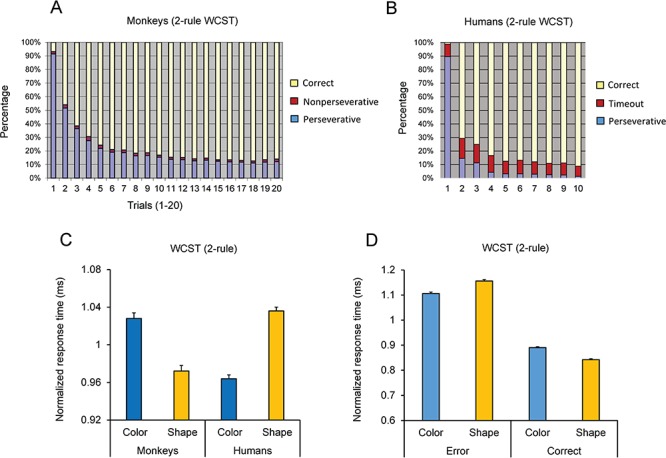
Performance of monkeys and humans in the 2-rule version of the WCST. (*A*) The mean percentage of correct, perseverative, and non-perseverative responses are shown in trials after the rule shift for 21 monkeys. Trial 1 is the first trial after the rule shift. Note that after the rule shift, monkeys’ correct responses dropped to a very low value; however, they adapted to the new rule achieved more than 70% correct in the fifth trial. Note that although a different sample was shown (until all the 36 samples were run) in each trial after the rule shift (no repetition of samples), the monkeys could shift to the new rule after a few trials. (*B*) The mean percentage of correct, perseverative, and time-out trials are shown in trials after the rule shift for 55 humans. (*C*) Normalized response time of correct responses was calculated in each subject by dividing the response time in each condition by the mean response time for all conditions in each subject. (*D*) Mean normalized response time is shown for perseverative and correct trials in monkeys performing the 2-rule version of the WCST. Response time was longer in perseverative error trials and shorter in correct trials when shape dimension was relevant. This led to a larger error slowing (difference in response time between error and correct trials) when shape was the relevant dimension. Error bars represent the standard error of the mean in all figures.

Macaque monkeys: in the data collection sessions, monkeys had to complete 300 trials of the 2-rule version of WCST (color and shape blocks; alternated within a session) in each daily session. Optimum performance in 300 trials could lead to the completion of a maximum of 15 blocks and so 14 rule-shifts (i.e., attaining the shift criterion of 17 corrects in 20 consecutive trials in 15 consecutive blocks reversing between color and shape dimensions). The mean number of dimension shifts was 10.84 ± 0.7 (Mean ± SE) in the 21 monkeys. In the 2-rule version of the WCST, responses could be classified as correct (matching by the relevant dimension), perseverative (matching by the alternative dimension), and nonperseverative error (selecting the test item that did not match the sample by color or shape dimension). The majority of errors was perseverative errors and mainly committed after the dimension shift (Fig. 2*A*). In the first trial after the dimension shift, the percentage of correct responses was 6.86 ± 1.75, which indicates that in the first trial after the unannounced dimension shift, in more than 90% of trials, the monkeys responded based on the previous dimension that was classified as perseverative response (Fig. 2*A*). This indicates that the monkeys were unaware of the dimension shift, and their behavior was efficiently governed by the selective attention to the relevant dimension in the block.

Humans: all 55 human participants successfully completed 8 blocks in the 2-rule version of WCST (4 color and 4 shape blocks; alternated within a session) in each testing session. Figure 2*B* shows the response distribution of human participants in 10 trials after the dimension shift (note that the criterion for dimension shift was 9 corrects out of 10 consecutive trials). To encourage speeded responses, a shorter response window was used for humans, and therefore, in some trials, there was no response in the available response window (timeout trials); humans’ errors were mainly composed of perseverative or timeout errors.

**Figure 3:**
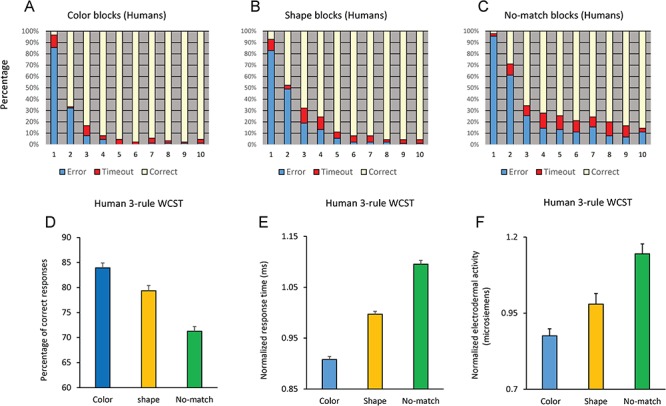
Performance of humans in the 3-rule version of the WCST**.** (*A*–*C*) Mean percentage of responses are shown in 10 trials after the rule shift when color (*A*), shape (*B*), or no-match (*C*) rule was relevant. (*D*) Mean percentage of correct responses when color, shape or no-match rule was relevant. (*E*) Mean-normalized response time in correct trials when color, shape, or no-match rule was relevant. (*F*) Mean-normalized EDA is shown when participants implemented color, shape, and no-match rules. *P* values for the pairwise comparisons of color and shape dimensions (paired *t-*test) are shown.

We also examined another cohort of 30 humans in a 3-rule version of the WCST (using the same sample set and rule-shift contingencies). In the 3-rule version, color-match, shape-match, and no-match rule could be used for selecting the target test item (Fig. 1). No-match rule required selecting the test item that did not match the sample by either color or shape dimension. Figure 3*A*–*C* shows the performance of humans in the first 10 trials after the rule shift in color-matching, shape-matching, and no-match blocks.

### Performance of Human Participants Differed Depending on the Relevant Dimension

 Figure 3*D* shows the mean percentage of correct responses with each 1 of the 3 rules in the 30 humans performing the 3-rule version of the WCST. A one-way repeated-measure ANOVA applied to the percentage of correct responses showed a significant main effect of Rule (F(2,58) = 58.73; *P* < 0.001) (Partial eta squared = 0.67). A pairwise comparison of performance (Bonferroni adjusted for multiple comparison) indicated a significant difference between color-matching and shape-matching rules (*P* = 0.002), between color-matching and no-match (*P* < 0.001) and between shape-matching and no-match (*P* < 0.001) rules. We also examined participants’ response time in correct trials when they applied each relevant rule. Response time was calculated as the time between the onset of test items and the first touch of the screen and reflects the time spent for resolving the conflict between potential dimensions and selecting the target item. In each participant, we first normalized the response time in each dimension by dividing it by the mean of response time averaged over color-matching, shape-matching, and no-match rules. Figure 3*E* shows that the response time in correct trials was the longest with no-match rule and the shortest with the color-matching rule. A repeated-measure ANOVA applied to the response time in correct trials showed a significant main effect of Rule (F(2,58) = 151.54; *P* < 0.001) (Partial eta squared = 0.84). A pairwise comparison of performance (Bonferroni adjusted for multiple comparison) indicated a significant difference between color and shape rules (*P* = 0.001), between color and no-match (*P* = 0.0001), and between shape and no-match (*P* = 0.001) rules. These findings indicate that for humans, no-match rule was the most difficult. Matching based on shape dimension was significantly more difficult than matching by color, and this difference in difficulty influenced both the percentage of correct responses and the response time. We also examined whether such a bias to color dimension was seen when humans performed the 2-rule version of the WCST. Performance of humans in the 2-rule version of the WCST was near ceiling, meaning that they immediately adapted to the new rule after each rule shift, and there was no significant difference in the percentage of correct responses between the color- and shape-matching rules (2-tailed paired *t-*test: *t*_54_ = 0.27; *P* = 0.79). However, response time was significantly shorter in color blocks (2-tailed paired *t-*test: *t*_54_ = 19.85; *P* = 0.001), indicating the higher speed of target selection in humans when they implemented the color-matching rule (Fig. 2*C*, Humans).

### Dimension-dependent Alterations in the EDA

We also examined whether the behavioral bias to color dimension as opposed to shape dimension was accompanied by task-related alterations in autonomic nervous system activity. Electrodermal signal (skin conductance) is associated with sympathetic nerve discharge. Previous studies (Bechara et al. 1999; Khalfa et al. 2002; Sequeira et al. 2009; Boucsein 2012) have shown alterations in EDA during cognitive task performance and particularly in relation to the behavioral outcome (Mansouri et al. 2017). We examined whether matching based on different rules influenced event-related EDA. A repeated-measure ANOVA [Rule (color/shape/no-match, within-subject factor] applied to the normalized EDA in color, shape, and no-match blocks showed a highly significant effect of Rule (F(2,48) = 13.15; *P* = 0.0001) (Partial eta squared = 0.35). Figure 3*F* shows that the mean normalized EDA was the lowest when participants were matching based on color dimension, however was at its highest level when no-match rule was applied. A planned comparison of EDA between color and shape dimensions indicated a significant difference (2-tailed *t-*test; *t*_24_ = 2.14, *P* = 0.04). This suggests that the bias to a particular dimension was accompanied by differences in the sympathetic nerve activity.

### Performance of Monkeys Differed Between Dimensions but the Direction of Difference Was Opposite to that of Humans

The superior performance of humans in implementing color dimension compared with shape dimension and the concomitant difference in EDA are intriguing considering that only simple shapes and colors were used for making the visual stimuli. Having observed such a significant bias in humans, we examined whether macaque monkeys showed the same behavioral bias to color dimension. Performance of monkeys were assessed in 15 sessions, and session means from each monkey were used as data points. The mean percentage of correct responses in color and shape blocks were 77.17 ± 0.27 and 76.95 ± 0.24, respectively. As was found in humans performing the 2-rule version of the WCST, monkeys did not have a significant difference in the percentage of correct responses between color-matching and shape-matching rules. We applied a 2-way ANOVA [Dimension (color/shape, within-subject factor) × Monkey (21 monkeys, between-subject factor)] to the percentage of correct responses. The main effect of dimension was not significant (F(1,292) = 0.35; *P* = 0.55).

However, there was a significant difference in monkeys’ response time between color and shape dimensions. We applied the 2-way ANOVA [Dimension (color/shape, within-subject factor) × Monkey (21 monkeys, between-subject factor)] to the normalized response time of correct responses. Response time was normalized in each monkey by dividing the response time in each dimension by the mean of response time averaged over color and shape dimensions. There was a highly significant main effect of dimension (F(1,292) = 137.25; *P* < 0.001) (Partial eta squared = 0.32). The response time was significantly “longer” when monkeys applied the color rule compared with the shape rule (Fig. 2*C*, Monkeys). This was in clear contrast to the findings in human participants where response time was significantly “shorter” when they applied the color rule (Fig. 2*C*, Humans).

We also directly compared the monkeys’ and humans’ dimension preference by applying a 2-way ANOVA [Species (monkeys/humans, between-subject factor) × Dimension (color/shape, within-subject factor)] to the mean normalized response time in correct trials in each subject. The main effect of Dimension was not significant as expected because the direction bias differed between humans and monkeys. However, there was a highly significant interaction between Species and Dimension factors (F(1,74) = 76.94; *P* < 0.0001) (Partial eta squared = 0.51). Figure 2*C* shows that monkeys and humans performed significantly faster with one of the dimensions; however, these species had an opposite preferences/advantages in matching based on color and shape dimensions.

**Figure 4:**
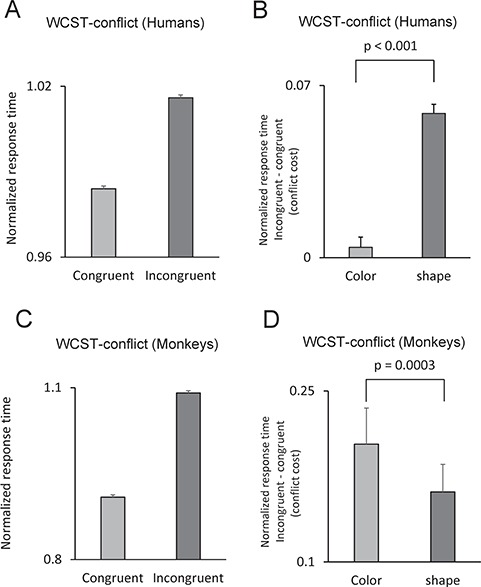
Conflict-induced behavioral adjustments were dependent on the relevant dimension. *A*–*D* show performance of humans and monkeys in a 2-rule version of the WCST in which the conflict between dimensions were modified trial by trial by running congruent and incongruent trials. (*A*) Mean-normalized response time of humans in congruent and incongruent conditions. Response time was longer in incongruent conditions, indicating that conflict between dimensions adversely affected humans’ performance. (*B*) The conflict cost (difference in response time between incongruent and congruent conditions) was significantly larger in humans when shape was the relevant dimension. (*C*) Response time was longer in incongruent conditions, indicating that conflict between dimensions adversely affected monkeys’ performance. (*D*) The conflict cost was significantly larger in monkeys when color was the relevant dimension.

### Conflict Cost Was Dependent on the Sensory Dimension

The presence of conflict in information processing adversely affects performance in terms of accuracy and response time (conflict cost) (Botvinick et al. 2001; Mansouri et al*.* 2017). Considering the attentional bias in humans to color dimension and in monkeys to shape dimension, we examined whether the conflict cost was dependent on the dimension. Thirteen monkeys and 55 humans were tested in a 2-rule version of the WCST in which the level of conflict between dimensions (color and shape) changed trial by trial (WCST-conflict). Congruent and incongruent trials were randomly intermixed throughout the testing period. Having observed the differences in participants’ EDA and response time between color and shape dimensions, we hypothesized that if the bias to a particular sensory dimension results from a general change in arousal/motivation, then there would be a uniform change in performance in both congruent and incongruent conditions, and therefore the conflict cost (difference between incongruent and congruent conditions) would remain unaltered. However, if conflict cost differs depending on the relevant dimension, it would indicate that resolving the conflict–competition between the 2 sensory dimensions was dependent on the relevant dimension.

We first applied a 2-way repeated-measure ANOVA [Dimension (color/shape, within-subject factor) × Conflict (incongruent/congruent, within-subject factor)] to the response time in humans. ANOVA showed that there was a highly significant main effect of dimension (F(1,54) = 221.58; *P* < 0.001) (Partial eta squared = 0.80) and a significant main effect of Conflict (F(1,54) = 127.96; *P* < 0.001) (Partial eta squared = 0.70) (Fig. 4*A*). Importantly, there was a highly significant interaction of Dimension and Conflict (F(1,54) = 120.13; *P* < 0.001) (Partial eta squared = 0.69), indicating that the effect of conflict on response time was dependent on the relevant dimension. In humans, conflict cost was significantly larger in shape blocks (Fig. 4*B*). In monkeys, the data with the WCST-conflict were collected in 13 monkeys in multiple sessions. We applied a 3-way repeated-measure ANOVA [Dimension (color/shape, within-subject factor) × Conflict (incongruent/congruent, within-subject factor) × Monkey (individual monkeys, between-subject factor)] to the mean response time in each session. There was a significant main effect of Dimension (F(1,121) = 48.01; *P* < 0.001) (Partial eta squared = 0.28) and a highly significant main effect of Conflict (F(1,54) = 402.83; *P* < 0.001) (Partial eta squared = 0.77), indicating that conflict significantly increased the response time in incongruent trials (Fig. 4*C*). Importantly, there was a significant interaction between Dimension and Conflict (F(1,54) = 12.04; *P* = 0.001) (Partial eta squared = 0.09), indicating that the effects of conflict were dependent on the relevant dimension. In monkeys the conflict cost was significantly larger when color was the relevant dimension (Fig. 4*D*).

**Figure 5:**
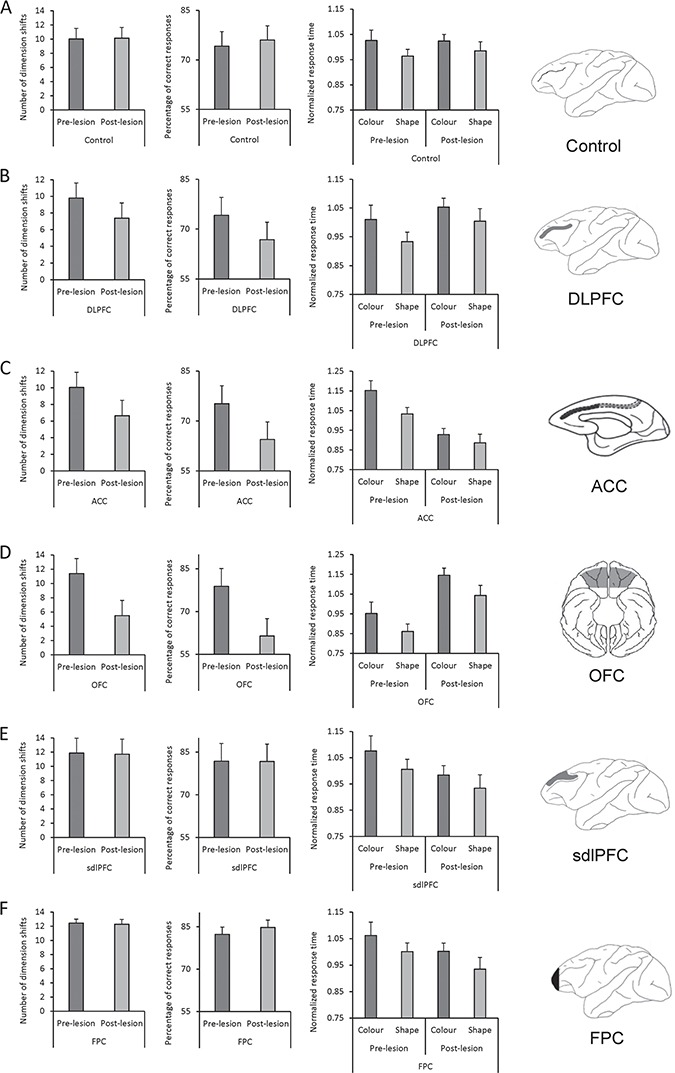
The effects of lesion in prefrontal and medial frontal regions on monkeys’ behavior. Left bar graphs in *A***–***F* show the mean number of rule shifts in prelesion and postlesion performance in Control monkeys (no lesion) and in monkeys with bilateral lesions in different cortical regions. Number of rule shifts in each session reflects the cognitive flexibility of monkeys in shifting between rules. There was no significant change in the number of rule shifts in Control (*A*), sdlPFC (*E*), and Frontopolar cortex (*F*) groups; however, it significantly decreased in monkeys with lesions in the DLPFC (*B*), ACC (*C*), and OFC (*D*) in postlesion testing. Middle bar graphs in *A–F* show the mean percentage of correct trials in prelesion and postlesion performance in Control monkeys and in monkeys with bilateral lesions in different cortical regions. There was no significant change in the percentage of correct trials in Control, sdlPFC, and FPC monkeys; however, it significantly decreased in monkeys with lesions in the DLPFC, ACC, and OFC in postlesion testing. Right bar graphs in *A–F* show the mean-normalized response time in correct trials in Control monkeys (*A*) and in monkeys with bilateral lesions in different cortical regions when color or shape dimension was relevant. Response time was longer when color was the relevant dimension in prelesion and postlesion testing for all groups. Although monkeys with lesions within DLPFC, in ACC, or in OFC showed deficits in cognitive flexibility to shift between dimensions, their attentional bias to shape dimension remained in the postlesion testing. Rightmost panels in *A–F* show the intended lesion extent in gray color on the schematic diagrams of macaque brain in monkeys with lesions in the DLPFC (Principal sulcus lesion: PS) (*B*), in the ACC (*C*), in the OFC (*D*), within the sdlPFC (superior part of DLPFC) (*E*), and in the frontopolar cortex (FPC) (*F*). All lesions were bilateral. The number of monkeys used in each group was as follows: Control (*n* = 6), DLPFC (*n* = 4), ACC (*n* = 4) (Buckley et al. 2009); sdlPFC (n = 3), OFC (n = 3) (Buckley et al. 2009; Mansouri et al. 2014); frontopolar cortex (*n* = 4) (Mansouri et al. 2015).

### Slowing in Perseverative Trials Was Dependent on the Visual Dimension

Almost all of the perseverative errors committed by humans occurred immediately after the rule shift, and these errors were inevitable errors because of the unannounced dimension change. However, monkeys committed occasional perseverative errors throughout the block, and therefore, we compared response time in perseverative and correct trials to assess whether response time was dimension-dependently modulated in error and correct trials. A 3-way repeated-measure ANOVA [(Response-type (perseverative/correct, within-subject factor) × Dimension (color/shape, within-subject factor) × Monkey (individual monkeys, between-subject factor)] was applied to the normalized response time in correct and perseverative trials (excluding the first perseverative error trial). There was a significant main effect of Response-type, indicating that response time in the perseverative trials was longer than that in the correct trials (F(1, 20) = 1933.54; *P* < 0.001) (Partial eta squared = 0.87). Importantly, there was a significant interaction of Response-type and Dimension factors (F(1, 20) = 93.56; *P* < 0.001) (Partial eta squared = 0.24). When a 2-way repeated-measure ANOVA [Dimension (color/shape, within-subject factor) × Monkey (individual monkeys, between-subject factor)] was applied to the normalized response time in perseverative trials (excluding the first perseverative error trial), there was a significant main effect of Dimension (F(1, 20) = 26.53; *P* < 0.001) (Partial eta squared = 0.08). Monkeys were slower in perseverative trials in shape blocks (when they erroneously applied the color rule) than perseverative trials in color blocks (when they erroneously applied the shape rule). For monkeys, inhibition of the shape rule might have been more difficult than that of the color rule, and therefore, monkeys had a longer response time in perseverative trials when shape dimension was relevant.

### Effects of Selective Lesions in Prefrontal and Medial Frontal Brain Regions on Monkeys’ Bias to a Particular Sensory Dimension

To explore the neural substrate of monkeys’ bias to matching according to a rule applied within a particular sensory dimension, we examined the consequence of selective and bilateral lesions within prefrontal and medial frontal regions. We hypothesized that if the dimension-based bias results from top-down attentional modulations, then lesions of prefrontal or medial frontal cortical regions would eliminate the bias. After collecting prelesion data in the WCST, selective lesions were made in DLPFC (4 monkeys) or in ACC (4 monkeys), and after a rest period, the monkeys were tested in postlesion sessions with the WCST analog (Buckley et al. 2009). Six other monkeys with no lesion served as a Control group, and the performance of these 3 groups were assessed between preoperative and postoperative periods. Control group monkeys had the equivalent, in duration, rest period of 2 weeks imposed between their equivalent pre- and posttesting periods. In the second stage of the studies, these 6 control monkeys received lesions within superior dlPFC (sdlPFC: 3 monkeys) or OFC (OFC group: 3 monkeys) (Fig. 5) (Buckley et al. 2009; Mansouri et al. 2014). Four other monkeys received bilateral lesions within the frontopolar cortex (FPC group: 4 monkeys) (Mansouri et al. 2015), and their performance was compared between the pre- and post-lesion testing (Fig. 5). In the WCST, the number of dimension shifts attained in each session reflects the overall ability in shifting between dimensions. We compared the number of dimension shifts between the preoperative and postoperative performance in each group. There was no significant change in the number of dimension shifts between the preoperative and postoperative performance in Control group, in sdlPFC group, or in the FPC group. However, there was a significant decline in the number of dimension shifts (Fig. 5*B-D*) and in the percentage of correct trials (Fig. 5*B-D*) in the DLPFC, ACC, and OFC groups as reported previously (Buckley et al*.* 2009).

To examine whether brain lesions affected the bias of monkeys to a particular dimension, we applied a repeated-measure 3-way ANOVA [Lesion (pre/post, within-subject factor) × Dimension (color/shape, within-subject factor) × Group (Control/DLPFC/ACC/OFC/sdlPFC/FPC, between-subject factor)] to the monkeys’ mean response times. We hypothesized that the monkeys’ bias toward a particular dimension would appear as a significant effect of Dimension. However, if a lesion in a brain region changes the monkeys’ bias toward sensory dimensions, then it would appear as a significant interaction between Lesion, Dimension, and Group factors. Indeed, the main effect of Dimension was highly significant (F(1, 18) = 14.02; *P* = 0.001) (Partial eta squared = 0.44), indicating a highly significant dimension-based bias in monkeys (Fig. 5). In addition, there was a significant interaction of the Lesion and Group factors (F(5, 18) = 3.31; *P* = 0.027) (Partial eta squared = 0.48), indicating that the monkeys’ response time was influenced by the lesion; monkeys in OFC group became slower, but monkeys in the ACC became faster in the postlesion testing sessions (Buckley et al. 2009). Importantly, there was no significant interaction between Lesion, Dimension, and Group factors (F(5, 18) = 0.81; *P* = 0.56) (Partial eta squared = 0.18), indicating that lesions in DLPFC, ACC, sdlPFC, OFC, or FPC did not change the monkeys’ bias toward a particular sensory dimension (Fig. 5). Figure 5*B-D* show that lesions within DLPFC, ACC, and OFC significantly changed the monkeys’ ability to implement inter-dimensional set-shifting (Buckley et al. 2009); however, the monkeys’ bias to shape dimension remained after selective lesions in these brain regions and were seen in prelesion and postlesion testings in all groups (Fig. 5). These findings indicate that the monkeys’ bias toward a particular sensory dimension was not dependent on the integrity of prefrontal or medial frontal cortical regions.

## Discussion

### Neural Substrate and Underlying Mechanisms of Humans’ and Monkeys’ Bias Toward Particular Dimensions

In a computerized WCST analog with 2 or 3 matching rules, we assessed performance and response time and found that young adult humans (University students) showed a highly significant behavioral bias to color dimension when it was compared with shape dimension. Interestingly, participants’ EDA (a measure of sympathetic nervous system activity) significantly differed between color and shape matching and reflected the aforementioned behavioral bias toward a particular sensory dimension (Fig. 3*F*). Such task-related alterations in the EDA might reflect changes in the arousal or emotional state of the participants in relation to the uncertainty or cognitive challenge/difficulty in processing a particular sensory dimension (Bechara et al*.* 1999; Khalfa et al*.* 2002; Sequeira et al*.* 2009; Boucsein 2012; Mansouri et al*.* 2017). Considering the dimension-dependent differences in participants’ EDA and response time, it might be assumed that participants’ arousal level differed between color and shape dimensions, and such arousal difference caused a nonspecific and uniform elevation in performance when implementation and shifting to color dimension was required. However, our findings do not support this hypothesis. We examined the behavior of humans and monkeys in response to different levels of conflict–competition between dimensions. The conflict cost was observed in response time of both species. If the behavioral bias to a particular sensory dimension was resulting from a general change in arousal, it should have affected the response time in both congruent and incongruent conditions, and therefore, conflict cost should have remained the same in color and shape blocks. However, in humans and monkeys, conflict cost was significantly larger when shape or color dimension was relevant, respectively (Figs 3*B* and 4*E*). The dimension dependency of conflict cost suggests that for humans, there was a behavioral bias toward color dimension, and therefore, resolving the competition between dimensions was more difficult when they had to inhibit the color dimension (Fig. 4*B*). In contrast, monkeys showed a behavioral bias toward shape dimension, and therefore, resolving the competition was more difficult when they had to inhibit the shape dimension (Fig. 4*D*). Examining monkeys’ performance in error trials also indicated that when shape was the relevant dimension, response time was shorter in correct trials but longer in error trials (in comparison to when color was the relevant dimension) (Fig. 2*D*). This also suggests that the bias in monkeys’ behavior was not arising from a nonspecific factor and instead reflected the influence of the relevant dimension on monkeys’ ability to inhibit the irrelevant dimension and shift between dimensions. Therefore, the dimension-dependent alterations in EDA likely reflected the cognitive challenge (difficulty) of matching based on a particular dimension (Fig. 3*F*).

In the context of an associative learning task, it has previously been reported that monkeys showed the same level of learning when they learned visual stimulus–reward association in color or shape dimensions (Baxter and Gaffan 2007). However, when the dimension changed, monkeys had more difficulty in shape discriminations if they had been doing color discriminations, but the same difficulty was not seen in animals shifting from shape to color (Baxter and Gaffan 2007). This difficulty in shape discrimination after a shift from color to shape dimension in the aforementioned study appears to contrast that observed in our study, namely an advantage in our monkeys for discrimination and matching-to-shape compared with matching-to-color in the WCST analog. However, this study (Baxter and Gaffan 2007) differed in various aspects from the WCST analog. In the associative learning task, the objects are repeatedly shown until associations are formed between particular exemplars of each dimension and the reward. Associative learning might be acquired through repetitive matching based on components or parts of objects and may not necessarily reflect an attentional set formation or shifting between abstract dimensions, as is required in the WCST analog (Baxter and Gaffan 2007). In the WCST analog used in our study, the sample and tests items and their locations were randomly chosen in each trial, and there was no repetition of samples following a dimension shift until all 36 samples were shown. Yet in just a few trials after the change, the monkeys nonetheless successfully shifted their matching performance to an alternate dimension and applied it to other exemplars in the new dimension (Fig. 2*A*). Therefore, the rapid rate of shift over of the monkeys’ performance from one rule to another after the dimension shift cannot be explained by within-block associative learning (stimulus–reward or position–reward associations) and instead suggests a highly efficient implementation and shift of attention between dimensions. Our previous studies also confirmed that monkeys could generalize the abstract dimension-based performance rules to novel exemplars (Mansouri and Tanaka 2002). Therefore, different mechanisms might underlie monkeys’ biases in object-based associative learning tasks and dimension-based behavior.

The difference in dimension-based bias between humans and monkeys might be attributed to differences in the structural and functional architecture of visual system and/or in selective attention to visual dimensions. In the WCST, selective attention to a dimension and shift to the other one is necessary, and therefore the observed behavioral advantage with a particular dimension might reflect the advantages in selective attention and its shift to that particular dimension (top-down processes). Alternatively, the bias to color or shape dimension might arise from differences in the lower stages of visual information processing (bottom-up processes). Previous studies have indicated similarities and also differences in the neural substrate and functional organization of visual system between humans and monkeys (Tanaka 1997; Van Essen et al*.* 2001; Orban et al*.* 2004; Matsuno and Fujita 2009). These studies have shown that humans and monkeys have a similar trichromatic color vision, visual acuity (Weinstein and Grether 1940; Cowey and Ellis 1967; De Valois et al. 1974; Kalloniatis and Harwerth 1991), and sensitivity to the temporal properties of visual stimuli (Matsuno and Fujita 2009). Monkeys and humans show similar abilities in object recognition (Tanaka 1997; Rajalingham et al. 2015) and even in perceiving visual illusions (Fujita 1997). Considering these findings, it is unlikely that monkeys’ bias toward shape dimension resulted from monkeys’ difficulty to detect and process color information or a better ability in shape discrimination.

The different nature of the dimension-based bias between humans and monkeys we report here might arise from functional differences in the architecture of attentional control in humans and monkeys (Patel et al. 2015). In humans, a dorsal attention network (frontoparietal network) is recruited during top-down control of attention to selectively focus attention on a particular goal-relevant aspect of sensory stimuli (Corbetta and Shulman 2002; Salo et al. 2017). In a functional MRI (fMRI) study, 2 macaque monkeys and 8 humans performed a task in which they had to detect a previously memorized item among serially presented visual images. Activation of frontoparietal regions were seen in both species; however, during target detection, activations in temporoparietal junction were observed in humans but not in monkeys, which suggested the absence of a functional ventral attention network in macaque monkeys (Patel et al*.* 2015). Other studies have also indicated that overt attention in monkeys and humans shows sensitivity to low-level features of the visual images such as luminance–contrast, salience, and texture–contrast; however, monkeys appear to be more sensitive to local changes, whereas humans express more consistency in selecting the focus of their attention (De Lillo et al. 2005; Einhauser et al. 2006). Humans and monkeys also show differences in focusing on global versus local features of visual stimuli (Fagot and Deruelle 1997; Hopkins and Washburn 2002; De Lillo et al*.* 2005), which indicate that in contrast to humans, monkeys are more focused on local features rather than global features. In our study, the visual stimuli were simple shapes, and therefore, there was little difference between the local and global features of the visual stimuli: however, it is still possible that monkeys’ attention on the local features, such as edges, led to a bias toward shape dimension compared with color in the simple images we used.

In the WCST analog, sensory dimensions such as color or shape matching are abstract rules because they can be applied to different exemplars, and subjects are supposed to keep the relevant matching rule in working memory and direct and shift selective attention to the relevant dimension accordingly (Mansouri et al*.* 2007; Mansouri et al*.* 2009). Our findings have indicated that both humans and monkeys implement and shift between these abstract dimensions (Mansouri and Tanaka 2002; Mansouri et al*.* 2007; Mansouri et al*.* 2009). Fize et al. (2011) compared performance of monkeys and humans in the context of a scene categorization task in which they had to categorize objects to animal/non-animals within different background images. They found that, like humans, monkeys successfully categorized the visual items, and the congruency between the background image and the object enhanced their performance. The authors also suggested, therefore, that monkeys have the capability for higher-order categorization of visual objects at abstract level. Similarly, Minamimoto et al. 2010 trained monkeys to respond to the color change of a visual-response cue. The category of objects (such as dogs or cats) in the background image shown behind the response cue indicated characteristics of the reward at the end of the trial (high-incentive or low-incentive cues). They found that monkeys rapidly learned to dissociate the visual categories and even generalized them to the new exemplars. Interestingly, a large lesion within the lateral prefrontal cortex did not impair the ability of monkeys to perform depending on learned categories or to learn new categories. These findings indicated that perceptual categorization of visual stimuli did not depend on the integrity of prefrontal cortex and might have been mediated through earlier stages of visual information processing.

### The Role of Prefrontal and Medial Frontal Cortical Areas in Formation, Bias, and Shift Between Sensory Dimensions in Attention-shifting Tasks

Previous electrophysiology studies have shown encoding of abstract rules (Mansouri et al. 2006; Mansouri et al. 2007, 2014) and object categories (Freedman 2001; Freedman and Assad 2016) in the activity of the prefrontal cortex neurons. While such a rich representation might imply that dimensions and categories emerge in these cortical areas, lesion studies to-date do not necessarily indicate an essential role of prefrontal cortical areas in the emergence of such dimensions or categories. Lesions in prefrontal cortex did not abolish learning and maintaining object categories (Minamimoto et al*.* 2010). These studies suggest that dimensions and categories might be represented in the prefrontal and medial frontal regions but do not necessarily emerge in these brain regions or depend on their integrity.

Previous studies indicate that DLPFC, OFC, and ACC are crucial for cognitive flexibility and executive control of goal-directed behavior (Miller and Cohen 2001; Mansouri et al*.* 2009; Mansouri et al*.* 2017). Activation in prefrontal and medial frontal cortical regions has been seen in various tasks requiring selective attention (Corbetta and Shulman 2002; Paneri and Gregoriou 2017; Salo et al*.* 2017). Our findings indicate that bilateral and selective lesions within the DLPFC, OFC, or ACC, all impaired monkeys’ ability to shift between sensory dimensions; however, it did not eliminate the bias toward a particular sensory dimension. Lesions within sdlPFC or frontopolar cortex were also ineffective in affecting monkeys’ performance, or their biases, in the WCST analog. The existence of biases to visual dimensions (color vs. shape) after bilateral prefrontal lesions indicates that the emergence of dimensions and the bias toward a particular dimension do not depend on the integrity of the prefrontal cortex.

**Figure 6:**
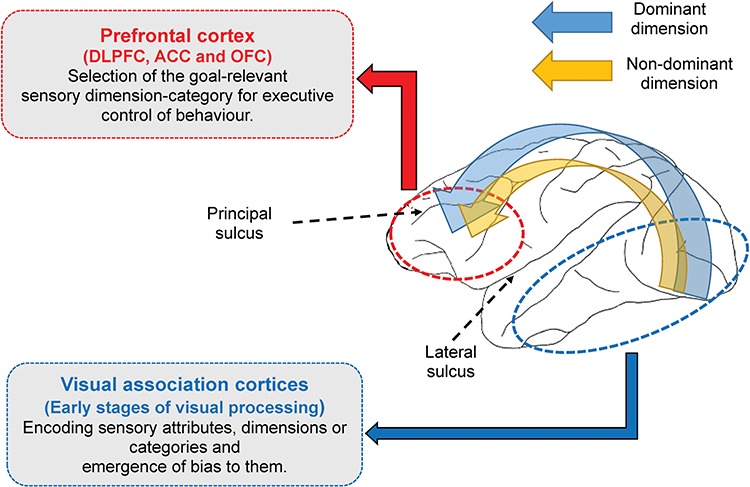
A model describing the role of prefrontal cortical regions in the formation of visual dimensions and goal-relevant shifts between them. We propose that the encoding and formation of dimensions and categories and the behavioral bias toward them emerge at earlier stages of visual information processing, without a necessary involvement of prefrontal cortical regions; however, goal-dependent shifts between sensory dimensions and categories require top-down modulation and depend on the integrity of prefrontal cortex.

## Conclusion

Our studies in monkeys in the context of an attentional set-shifting task (WCST analog) have led to 2 key findings: lesions in the prefrontal and medial frontal cortical regions do not abolish performance biases to sensory dimension (e.g., color vs. shape); however, they impair goal-dependent shifts between these dimensions (Buckley et al*.* 2009; Mansouri et al. 2009; Mansouri et al*.* 2017). Future studies need to examine whether the dimension-based bias in rule-guided behavior in humans and monkeys appears in other cognitive domains and investigate the underlying neurobiological substrate for any species differences. It is important to examine whether such differences also exist between humans and other anthropoid apes (such as chimpanzees) because that would bring further insight to the functional milestones in the development of the anthropoid brain. There may even be differences between different species of macaque. Differences in ecological adaptations and social behaviors are known to exist between the 2 monkey species we investigated (mulatta and fuscata) (Owren and Dieter, 1989; Owren et al. 1993; Chu et al. 2007), which may lead to the emergence of different strategies in achieving goals. However, considering the ethical requirement to reduce the number of animals enrolled in neuroscience research, it was not possible to have sufficiently large sample sizes available to enable comparisons of lesion effects between the two macaque species in this study.

Based on our findings and previous studies examining the neural substrate of visual categorization (Freedman 2001; Minamimoto et al*.* 2010; Freedman and Assad 2016), we propose that goal-dependent shifts between sensory dimensions and categories might require top-down modulation, such that monkeys with prefrontal and medial frontal cortical lesions show deficits in shifting between dimensions (Fig. 6). However, formation and representation of dimensions and categories and the behavioral bias toward a particular dimension might emerge at earlier stages of visual information processing, without necessary involvement of top-down modulation (Fig. 6); accordingly, lesions in the prefrontal and medial frontal cortex do not affect their occurrence. The alteration of such biases in neurodevelopmental disorders (Supplementary material) necessitates a better understanding of their underlying mechanisms.

## References

[ref1] Baxter MG, Gaffan D 2007 Asymmetry of attentional set in rhesus monkeys learning colour and shape discriminations. Q J Exp Psychol. 60:1–8. 10.1080/17470210600971485 PMC1764629 17162503

[ref2] Bechara A, Damasio H, Damasio AR, Lee GP 1999 Different contributions of the human amygdala and ventromedial prefrontal cortex to decision-making. J Neurosci. 19:5473–5481. 10377356 10.1523/JNEUROSCI.19-13-05473.1999 PMC6782338

[ref3] Boschin EA, Brkic MM, Simons JS, Buckley MJ 2017a Distinct roles for the anterior cingulate and dorsolateral prefrontal cortices during conflict between abstract rules. Cereb Cortex. 27:34–45. 28365775 10.1093/cercor/bhw350 PMC5939207

[ref4] Boschin EA, Mars RB, Buckley MJ 2017b Transcranial magnetic stimulation to dorsolateral prefrontal cortex affects conflict-induced behavioural adaptation in a Wisconsin Card Sorting Test analogue. Neuropsychologia. 94:36–43. 27889392 10.1016/j.neuropsychologia.2016.11.015 PMC5226064

[ref5] Botvinick MM, Braver TS, Barch DM, Carter CS, Cohen JD 2001 Conflict monitoring and cognitive control. Psychol Rev. 108:624–652. 11488380 10.1037/0033-295x.108.3.624

[ref6] Boucsein W 2012 Electrodermal activity. Boston, (MA): Springer US

[ref7] Brown AL, Campione JC 1971 Colour dominance in preschool children as a function of specific cue preferences. Child Dev. 42:1495. 5156649

[ref8] Buckley MJ, Mansouri FA, Hoda H, Mahboubi M, Browning PGF, Kwok SC, Phillips A, Tanaka K 2009 Dissociable components of rule-guided behaviour depend on distinct medial and prefrontal regions. Science. 325:52–58. 19574382 10.1126/science.1172377

[ref9] Chu JH, Lin YS, Wu HY 2007 Evolution and dispersal of three closely related macaque species, Macaca mulatta, M. cyclopis, and M. fuscata, in the eastern Asia. Mol Phylogenet Evol. 43:418–429. 17321761 10.1016/j.ympev.2006.11.022

[ref10] Corbetta M, Shulman GL 2002 Control of goal-directed and stimulus-driven attention in the brain. Nat Rev Neurosci. 3:201–215. 11994752 10.1038/nrn755

[ref11] Cowey A, Ellis CM 1967 Visual acuity of rhesus and squirrel monkeys. J Comp Physiol Psychol. 64:80–84. 4965051 10.1037/h0024821

[ref12] De Lillo C, Spinozzi G, Truppa V, Naylor DM 2005 A comparative analysis of global and local processing of hierarchical visual stimuli in young children (Homo sapiens) and monkeys (Cebus apella). J Comp Psychol. 119:155–165. 15982159 10.1037/0735-7036.119.2.155

[ref13] De Valois RL, Morgan HC, Polson MC, Mead WR, Hull EM 1974 Psychophysical studies of monkey vision—I. Macaque luminosity and colour vision tests. Vision Res. 14:53–67. 4204837 10.1016/0042-6989(74)90116-3

[ref14] Dias R, Robbins TW, Roberts AC 1996 Primate analogue of the Wisconsin Card Sorting Test: effects of excitotoxic lesions of the prefrontal cortex in the marmoset. Behav Neurosci. 110:872–886. 8918991 10.1037//0735-7044.110.5.872

[ref15] Einhauser W, Kruse W, Hoffmann KP, Konig P 2006 Differences of monkey and human overt attention under natural conditions. Vision Res. 46:1194–1209. 16375943 10.1016/j.visres.2005.08.032

[ref16] Ellefson MR, Shapiro LR, Chater N 2006 Asymmetrical switch costs in children. Cogn Dev. 21:108–130.

[ref17] Fagot J, Deruelle C 1997 Processing of global and local visual information and hemispheric specialization in humans (Homo sapiens) and baboons (Papio papio). J Exp Psychol Hum Percept Perform. 23:429–442. 9104003 10.1037//0096-1523.23.2.429

[ref18] Fize D, Cauchoix M, Fabre-Thorpe M 2011 Humans and monkeys share visual representations. Proc Natl Acad Sci U S A. 108:7635–7640. 21502509 10.1073/pnas.1016213108 PMC3088612

[ref19] Freedman DJ 2001 Categorical representation of visual stimuli in the primate prefrontal cortex. Science. 291:312–316. 11209083 10.1126/science.291.5502.312

[ref20] Freedman DJ, Assad JA 2016 Neuronal mechanisms of visual categorization: an abstract view on decision making. Annu Rev Neurosci. 39:129–147. 27070552 10.1146/annurev-neuro-071714-033919

[ref21] Fujita K 1997 Perception of the Ponzo illusion by rhesus monkeys, chimpanzees, and humans: similarity and difference in the three primate species. Percept Psychophys. 59:284–292. 9055623 10.3758/bf03211896

[ref22] Grant DA, Curran JF 1952 Relative difficulty of number, form, and color concepts of a Weigl-type problem using unsystematic number cards. J Exp Psychol. 43:408–413. 14946354 10.1037/h0054914

[ref23] Grant DA, Jones OR, Tallantis B 1949 The relative difficulty of the number, form, and color concepts of a Weigl-type problem. J Exp Psychol. 39:552–557. 18140139 10.1037/h0062126

[ref24] Hopkins WD, Washburn DA 2002 Matching visual stimuli on the basis of global and local features by chimpanzees (*Pan troglodyte*s) and rhesus monkeys (*Macaca mulatta*). Anim Cogn. 5:27–31. 11957399 10.1007/s10071-001-0121-8 PMC2043159

[ref25] Kalloniatis M, Harwerth RS 1991 Effects of chromatic adaptation on opponent interactions in monkey increment-threshold spectral-sensitivity functions. J Opt Soc Am. 8:1818. 10.1364/josaa.8.001818 1744778

[ref26] Khalfa S, Isabelle P, Jean-Pierre B, Manon R 2002 Event-related skin conductance responses to musical emotions in humans. Neurosci Lett. 328:145–149. 12133576 10.1016/s0304-3940(02)00462-7

[ref27] Konishi S, Nakajima K, Uchida I, Kameyama M, Nakahara K, Sekihara K, Miyashita Y 1998 Transient activation of inferior prefrontal cortex during cognitive set shifting. Nat Neurosci. 1:80–84. 10195114 10.1038/283

[ref28] Kuwabara M, Mansouri FA, Buckley MJ, Tanaka K 2014 Cognitive control functions of anterior cingulate cortex in macaque monkeys performing a Wisconsin Card Sorting Test analog. J Neurosci. 34:7531–7547. 24872558 10.1523/JNEUROSCI.3405-13.2014 PMC4035517

[ref29] Mackey S, Petrides M 2010 Quantitative demonstration of comparable architectonic areas within the ventromedial and lateral orbital frontal cortex in the human and the macaque monkey brains. Eur J Neurosci. 32:1940–1950. 21050280 10.1111/j.1460-9568.2010.07465.x

[ref30] Mansouri FA, Acevedo N, Illipparampil R, Fehring DJ, Fitzgerald PB, Jaberzadeh S 2017a Interactive effects of music and prefrontal cortex stimulation in modulating response inhibition. Sci Rep. 7:18096. 29273796 10.1038/s41598-017-18119-x PMC5741740

[ref31] Mansouri FA, Buckley MJ, Mahboubi M, Tanaka K 2015 Behavioural consequences of selective damage to frontal pole and posterior cingulate cortices. Proc Natl Acad Sci U S A. 112:E3940–E3949. 26150522 10.1073/pnas.1422629112 PMC4517212

[ref32] Mansouri FA, Buckley MJ, Tanaka K 2007 Mnemonic function of the dorsolateral prefrontal cortex in conflict-induced behavioural adjustment. Science. 318:987–990. 17962523 10.1126/science.1146384

[ref33] Mansouri FA, Buckley MJ, Tanaka K 2014 The essential role of primate orbitofrontal cortex in conflict-induced executive control adjustment. J Neurosci. 34:11016–11031. 25122901 10.1523/JNEUROSCI.1637-14.2014 PMC4131015

[ref34] Mansouri FA, Egner T, Buckley MJ 2017b Monitoring demands for executive control: shared functions between human and nonhuman primates. Trends Neurosci. 40:15–27. 27986294 10.1016/j.tins.2016.11.001

[ref35] Mansouri FA, Fehring DJ, Feizpour A, Gaillard A, Rosa MG, Rajan R, Jaberzadeh S 2016a Direct current stimulation of prefrontal cortex modulates error-induced behavioral adjustments. Eur J Neurosci. 44:1856–1869. 27207192 10.1111/ejn.13281

[ref36] Mansouri FA, Fehring DJ, Gaillard A, Jaberzadeh S, Parkington H 2016b Sex dependency of inhibitory control functions. Biol Sex Differ. 7:11. 26862388 10.1186/s13293-016-0065-y PMC4746892

[ref37] Mansouri FA, Matsumoto K, Tanaka K 2006 Prefrontal cell activities related to monkeys’ success and failure in adapting to rule changes in a Wisconsin Card Sorting Test analog. J Neurosci. 26:2745–2756. 16525054 10.1523/JNEUROSCI.5238-05.2006 PMC6675148

[ref38] Mansouri FA, Tanaka K 2002 Behavioral evidence for working memory of sensory dimension in macaque monkeys. Behav Brain Res. 136:415–426. 12429403 10.1016/s0166-4328(02)00182-1

[ref39] Mansouri FA, Tanaka K, Buckley MJ 2009 Conflict-induced behavioural adjustment: a clue to the executive functions of the prefrontal cortex. Nat Rev Neurosci. 10:141–152. 19153577 10.1038/nrn2538

[ref40] Matsuno T, Fujita K 2009 A comparative psychophysical approach to visual perception in primates. Primates. 50:121–130. 19153806 10.1007/s10329-008-0128-8

[ref41] Maunsell JHR, Treue S 2006 Feature-based attention in visual cortex. Trends Neurosci. 29:317–322. 16697058 10.1016/j.tins.2006.04.001

[ref42] Miller EK, Cohen JD 2001 An integrative theory of prefrontal cortex function. Annu Rev Neurosci. 24:167–202. 11283309 10.1146/annurev.neuro.24.1.167

[ref43] Minamimoto T, Saunders RC, Richmond BJ 2010 Monkeys quickly learn and generalize visual categories without lateral prefrontal cortex. Neuron. 66:501–507. 20510855 10.1016/j.neuron.2010.04.010

[ref44] Monchi O, Petrides M, Petre V, Worsley K, Dagher A 2001 Wisconsin Card Sorting revisited: distinct neural circuits participating in different stages of the task identified by event-related functional magnetic resonance imaging. J Neurosci. 21:7733–7741. 11567063 10.1523/JNEUROSCI.21-19-07733.2001 PMC6762921

[ref45] Nakahara K 2002 Functional MRI of macaque monkeys performing a cognitive set-shifting task. Science. 295:1532–1536. 11859197 10.1126/science.1067653

[ref46] Orban GA, Van Essen D, Vanduffel W 2004 Comparative mapping of higher visual areas in monkeys and humans. Trends Cogn Sci. 8:315–324. 15242691 10.1016/j.tics.2004.05.009

[ref47] Otto W, Askov E 1968 The role of colour in learning and instruction. J Spec Educ. 2:155–165.

[ref48] Owren MJ, Dieter JA 1989 Infant cross-fostering between Japanese (Macaca fuscata) and rhesus macaques (M. mulatta). Am J Primatol. 18:245–250. 31964036 10.1002/ajp.1350180308

[ref49] Owren MJ, Dieter JA, Seyfarth RM, Cheney DL 1993 Vocalizations of rhesus (Macaca mulatta) and Japanese (M. fuscata) macaques cross-fostered between species show evidence of only limited modification. Dev Psychobiol. 26:389–406. 8270122 10.1002/dev.420260703

[ref50] Paneri S, Gregoriou GG 2017 Top-down control of visual attention by the prefrontal cortex. Functional specialization and long-range interactions. Front Neurosci. 11:545. 29033784 10.3389/fnins.2017.00545 PMC5626849

[ref51] Patel GH, Yang D, Jamerson EC, Snyder LH, Corbetta M, Ferrera VP 2015 Functional evolution of new and expanded attention networks in humans. Proc Natl Acad Sci U S A. 112:9454–9459. 26170314 10.1073/pnas.1420395112 PMC4522817

[ref52] Petrides M, Cadoret G, Mackey S 2005 Orofacial somatomotor responses in the macaque monkey homologue of Broca’s area. Nature. 435:1235–1238. 15988526 10.1038/nature03628

[ref53] Petrides M, Pandya DN 1999 Dorsolateral prefrontal cortex: comparative cytoarchitectonic analysis in the human and the macaque brain and corticocortical connection patterns. Eur J Neurosci. 11:1011–1036. 10103094 10.1046/j.1460-9568.1999.00518.x

[ref54] Petrides M, Tomaiuolo F, Yeterian EH, Pandya DN 2012 The prefrontal cortex: comparative architectonic organization in the human and the macaque monkey brains. Cortex. 48:46–57. 21872854 10.1016/j.cortex.2011.07.002

[ref55] Prevor MB, Diamond A 2005 Colour-object interference in young children: a Stroop effect in children 3(1/2)-6(1/2) years old. Cogn Dev. 20:256–278. 18079980 10.1016/j.cogdev.2005.04.001 PMC2134842

[ref56] Rajalingham R, Schmidt K, DiCarlo JJ 2015 Comparison of object recognition behaviour in human and monkey. J Neurosci. 35:12127–12136. 26338324 10.1523/JNEUROSCI.0573-15.2015 PMC4556783

[ref57] Salo E, Salmela V, Salmi J, Numminen J, Alho K 2017 Brain activity associated with selective attention, divided attention and distraction. Brain Res. 1664:25–36. 28363436 10.1016/j.brainres.2017.03.021

[ref58] Sequeira H, Hot P, Silvert L, Delplanque S 2009 Electrical autonomic correlates of emotion. Int J Psychophysiol. 71:50–56. 18723054 10.1016/j.ijpsycho.2008.07.009

[ref59] Stuss DT, Levine B, Alexander MP, Hong J, Palumbo C, Hamer L, Murphy KJ, Izukawa D 2000 Wisconsin Card Sorting Test performance in patients with focal frontal and posterior brain damage: effects of lesion location and test structure on separable cognitive processes. Neuropsychologia. 38:388–402. 10683390 10.1016/s0028-3932(99)00093-7

[ref60] Tanaka K 1997 Mechanisms of visual object recognition: monkey and human studies. Curr Opin Neurobiol. 7:523–529. 9287204 10.1016/s0959-4388(97)80032-3

[ref61] Van Essen DC, Lewis JW, Drury HA, Hadjikhani N, Tootell RBH, Bakircioglu M, Miller MI 2001 Mapping visual cortex in monkeys and humans using surface-based atlases. Vision Res. 41:1359–1378. 11322980 10.1016/s0042-6989(01)00045-1

[ref62] Vogt BA, Pandya DN, Rosene DL 1987 Cingulate cortex of the rhesus monkey: I. Cytoarchitecture and thalamic afferents. J Comp Neurol. 262:256–270. 3624554 10.1002/cne.902620207

[ref63] Weinstein B, Grether WF 1940 A comparison of visual acuity in the rhesus monkey and man. J Comp Psychol. 30:187–195.

[ref64] Yi L, Liu Y, Li Y, Fan Y, Huang D, Gao D 2012 Visual scanning patterns during the dimensional change card sorting task in children with autism spectrum disorder. Autism Res Treat. 2012:1–11. 10.1155/2012/123053 PMC3459256 23050145

